# *N*,*N*′-(4-Chloro­benzyl­idene)dipyrimidin-2-amine

**DOI:** 10.1107/S1600536809045243

**Published:** 2009-11-04

**Authors:** Masoumeh Tabatabaee, Leila Masoodpour, Mitra Gassemzadeh, Fatemeh Hakimi

**Affiliations:** a Department of Chemistry, Islamic Azad University, Yazd Branch, Yazd, Iran

## Abstract

The title compound, C_15_H_13_ClN_6_, contains two pyrimidine rings and one benzene ring, where the dihedral angle between the planes through the pyrimidine rings is 81.57 (10)°, and those between the pyrimidine rings and the benzene ring are 84.02 (8) and 89.46 (7)°, indicating that the three rings are almost perpendicular. In the crystal, inter­molecular N—H⋯N hydrogen bonds link the mol­ecules into infinite chains along (100).

## Related literature

For the biological activity of pyrimidine derivatives, see: Onal & Altral (1999 ▶); Ponticelli *et al.* (1999 ▶). For studies of the reactions of heterocyclic amines with aromatic aldehydes to prepare new ligands, see: Tabatabaee *et al.* (2006 ▶); Tabatabaee, Ghassemzadeh, Dehghan *et al.* (2007 ▶); Tabatabaee, Ghassemzadeh, Zarabi *et al.* (2007 ▶); Tabatabaee, Ghassemzadeh *et al.* (2008 ▶); Tabatabaee, Hakimi *et al.* (2008 ▶).
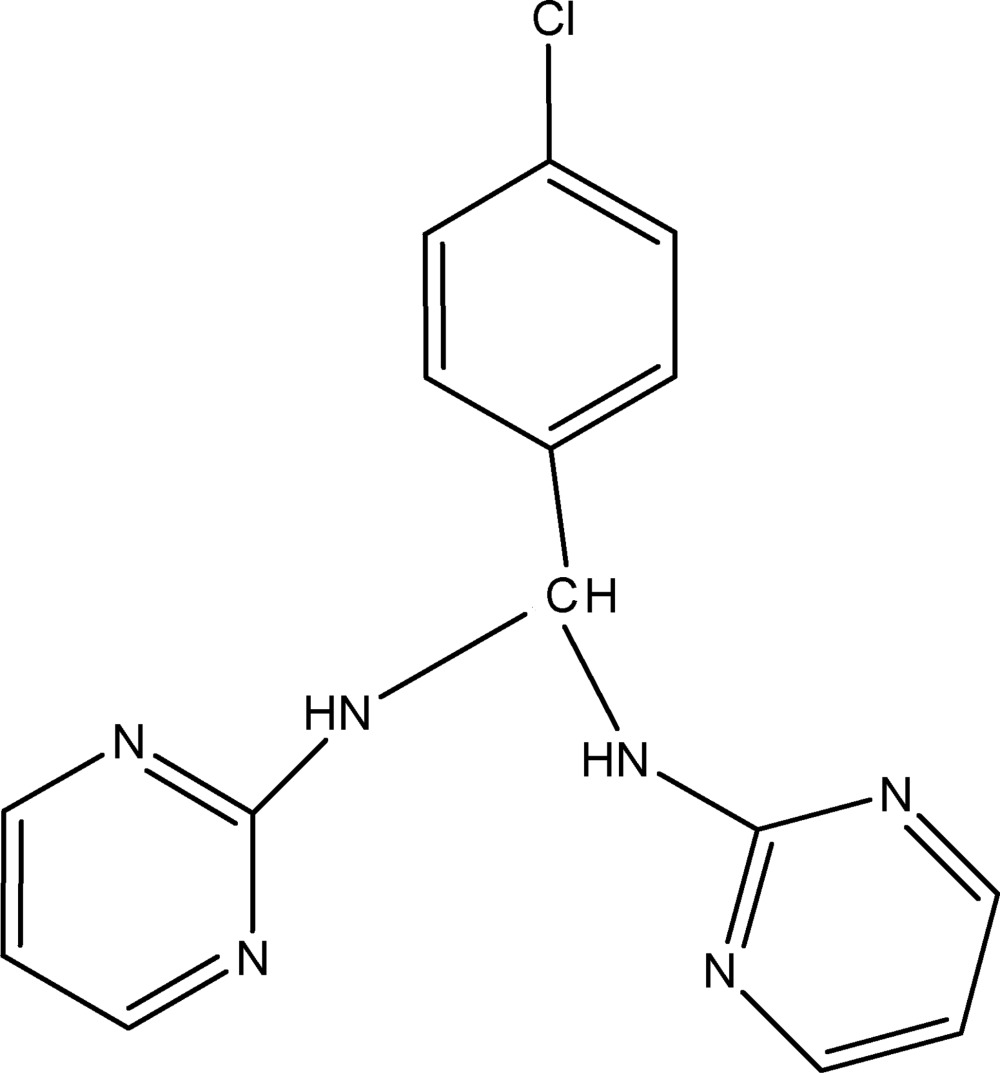


## Experimental

### 

#### Crystal data

C_15_H_13_ClN_6_
*M* *_r_* = 312.76
Monoclinic, 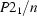

*a* = 9.6030 (14) Å
*b* = 10.5706 (15) Å
*c* = 14.792 (2) Å
β = 100.331 (3)°
*V* = 1477.2 (4) Å^3^
*Z* = 4
Mo *K*α radiation
μ = 0.26 mm^−1^
*T* = 120 K
0.17 × 0.15 × 0.14 mm

#### Data collection

Bruker SMART 1000 CCD area-detector diffractometer
Absorption correction: multi-scan (*SADABS*; Sheldrick, 1998 ▶) *T*_min_ = 0.950, *T*_max_ = 0.964
15899 measured reflections
3924 independent reflections
2429 reflections with *I* > 2σ(*I*)
*R*_int_ = 0.063

#### Refinement

*R*[*F* ^2^ > 2σ(*F* ^2^)] = 0.061
*wR*(*F* ^2^) = 0.163
*S* = 1.01
3924 reflections
207 parameters
H atoms treated by a mixture of independent and constrained refinement
Δρ_max_ = 0.55 e Å^−3^
Δρ_min_ = −0.28 e Å^−3^

### 

Data collection: *SMART* (Bruker, 1998 ▶); cell refinement: *SAINT-Plus* (Bruker, 1998 ▶); data reduction: *SAINT-Plus*; program(s) used to solve structure: *SHELXTL* (Sheldrick, 2008 ▶); program(s) used to refine structure: *SHELXTL*; molecular graphics: *SHELXTL*; software used to prepare material for publication: *SHELXTL*.

## Supplementary Material

Crystal structure: contains datablocks I, global. DOI: 10.1107/S1600536809045243/ez2187sup1.cif

Structure factors: contains datablocks I. DOI: 10.1107/S1600536809045243/ez2187Isup2.hkl

Additional supplementary materials:  crystallographic information; 3D view; checkCIF report

## Figures and Tables

**Table 1:** Hydrogen-bond geometry (Å, °)

*D*—H⋯*A*	*D*—H	H⋯*A*	*D*⋯*A*	*D*—H⋯*A*
N1—H1*N*⋯N3^i^	0.84 (3)	2.20 (3)	3.033 (3)	174 (3)
N4—H4*N*⋯N6^ii^	0.83 (3)	2.24 (3)	3.057 (3)	172 (3)

Symmetry codes: (i) 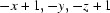; (ii) 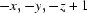.

**Fig. 1.**
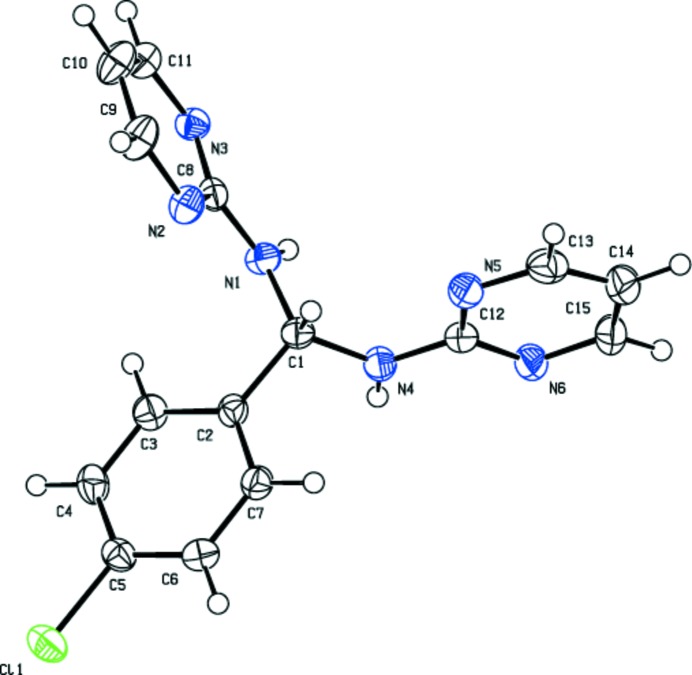
The molecular structure of (I). Displacement ellipsoids are drawn at the 50% probability level.

**Fig. 2.**
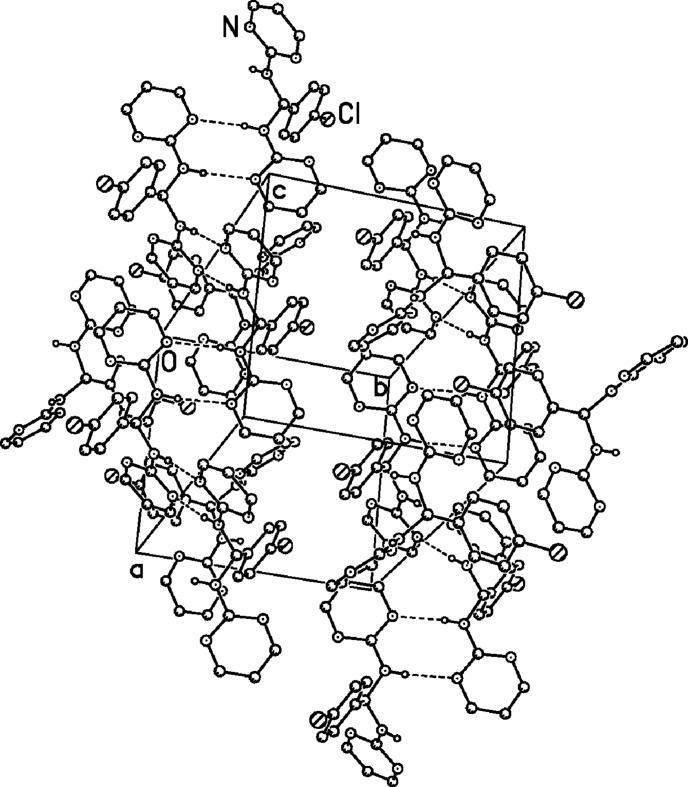
Packing diagram of (I), molecules are linked into infinite one dimensional chains by hydrogen-bond interactions (dashed lines).

**Fig. 3.**
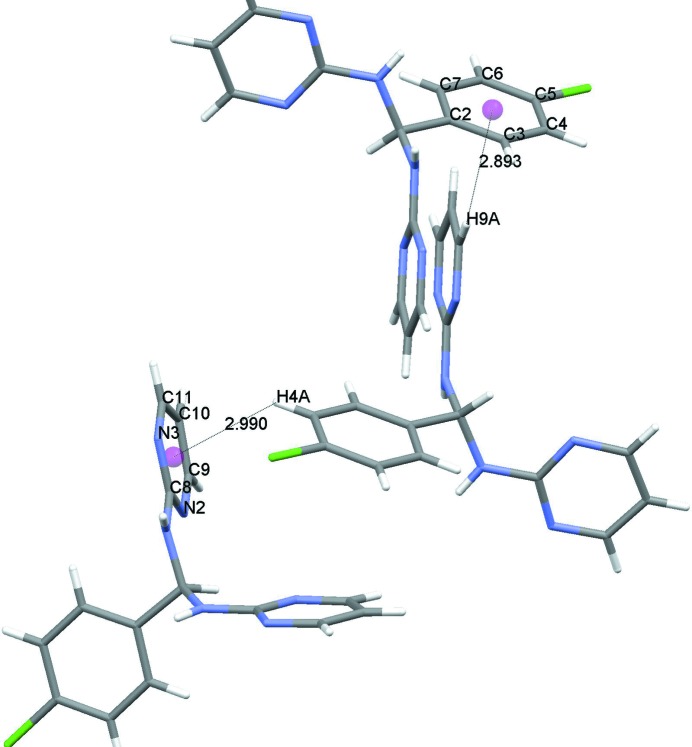
Intermolecular C—H···π interactions (dashed lines) between aromatic rings of adjacent molecules.

## supplementary crystallographic information

### Comment

Pyrimidine derivatives represent a class of heterocycles of great importance. Many pyrimidines, or their derivatives, possess remarkable biological activity and have been widely used in medicinal and industrial applications (Onal & Altral, 1999; Ponticelli *et al.*, 1999). In the continuation of our recent work on the reactions of heterocyclic amines with aromatic aldehydes to prepare new ligands (Tabatabaee *et al.*, 2006; Tabatabaee, Ghassemzadeh, Dehghan *et al.*, 2007; Tabatabaee, Ghassemzadeh, Zarabi *et al.*, 2007; Tabatabaee, Ghassemzadeh *et al.*, 2008; Tabatabaee, Hakimi *et al.*, 2008) we report our results on the reaction of 2-aminopyrimidine and 4-chlorobenzaldehyde in this communication.

The crystal structure of (I) (Fig. 1) shows that one molecule of 4-chlorobenzaldehyde reacted with two molecules of 2-aminopyrimidine to form (I). Bond lengths and angles are unexceptional. The compound contains two pyrimidine (A:N2/C8/N3/C11/C10/C9 and B: N5/C12/N6/C15/C14/C13) and one benzene (C: C2/C3/C4/C5/C6/C7) rings, The dihedral angles formed by the planes through A and B is 81.57 (10)°, through A and C is 84.02 (8)° and through B and C is 89.49 (7)°, indicating that the three rings are almost perpendicular.

Intermolecular N—H···N hydrogen bonds link the molecules into infinite one-dimensional chains along (100) (Table 1 and Fig 2). An interesting feature of compound (I) is the presence of C—H···π stacking interactions between C—H groups from one molecule and aromatic rings on adjacent molecules. The C—H···π distance is 2.89 Å for C9—H9A···*Cg*3 (*Cg*3: C2/C3—C7), with an angle of 133.21° and 2.99 Å for C4—H4A^···^*Cg*1 (*Cg*1: N2/C8 — C9) with an angle of 132.27° (Fig. 3).

### Experimental

A solution of 2-aminopyrimidine (0.951 g, 10 mmol) in EtOH (10 ml) was treated with 4- chlorobenzaldehyde (0.7 g, 5 mmol) and the resulting mixture was acidified with 37% hydrochloric acid (0.2 ml). The reaction mixture was refluxed for 12 h. The solid residue was filtered and the filtrate was kept at 293 K. Colorless crystals of the title compound were obtained after a few days (yield 92%).

### Refinement

The hydrogen atoms of NH groups were found in difference Fourier syntheses and refined isotropically. The H(C) atom positions were calculated and refined in isotropic approximation using a riding model with the *U*_iso_(H) parameters equal to 1.2 *U*_eq_(Ci), where U(Ci) are the equivalent thermal parameters of the CH and CH_2_ carbon atoms to which the corresponding H atoms are bonded.

### Crystal data

**Table:**

C_15_H_13_ClN_6_	*F*(000) = 648
*M**_r_* = 312.76	*D*_x_ = 1.406 Mg m^−^^3^
Monoclinic, *P*2_1_/*n*	Mo *K*α radiation, λ = 0.71073 Å
Hall symbol: -P 2yn	Cell parameters from 697 reflections
*a* = 9.6030 (14) Å	θ = 3–30°
*b* = 10.5706 (15) Å	µ = 0.26 mm^−^^1^
*c* = 14.792 (2) Å	*T* = 120 K
β = 100.331 (3)°	Prism, colorless
*V* = 1477.2 (4) Å^3^	0.17 × 0.15 × 0.14 mm
*Z* = 4	

### Data collection

**Table:**

Bruker SMART 1000 CCD area-detector diffractometer	3924 independent reflections
Radiation source: fine-focus sealed tube	2429 reflections with *I* > 2σ(*I*)
graphite	*R*_int_ = 0.063
ω scans	θ_max_ = 29.0°, θ_min_ = 2.4°
Absorption correction: multi-scan (*SADABS*; Sheldrick, 1998)	*h* = −13→13
*T*_min_ = 0.950, *T*_max_ = 0.964	*k* = −14→14
15899 measured reflections	*l* = −20→20

### Refinement

**Table:**

Refinement on *F*^2^	Primary atom site location: structure-invariant direct methods
Least-squares matrix: full	Secondary atom site location: difference Fourier map
*R*[*F*^2^ > 2σ(*F*^2^)] = 0.061	Hydrogen site location: mixed
*wR*(*F*^2^) = 0.163	H atoms treated by a mixture of independent and constrained refinement
*S* = 1.01	*w* = 1/[σ^2^(*F*_o_^2^) + (0.061*P*)^2^ + 1.85*P*] where *P* = (*F*_o_^2^ + 2*F*_c_^2^)/3
3924 reflections	(Δ/σ)_max_ = 0.001
207 parameters	Δρ_max_ = 0.55 e Å^−^^3^
0 restraints	Δρ_min_ = −0.28 e Å^−^^3^

### Special details

**Table:**

Geometry. All e.s.d.'s (except the e.s.d. in the dihedral angle between two l.s. planes) are estimated using the full covariance matrix. The cell e.s.d.'s are taken into account individually in the estimation of e.s.d.'s in distances, angles and torsion angles; correlations between e.s.d.'s in cell parameters are only used when they are defined by crystal symmetry. An approximate (isotropic) treatment of cell e.s.d.'s is used for estimating e.s.d.'s involving l.s. planes.
Refinement. Refinement of *F*^2^ against ALL reflections. The weighted *R*-factor *wR* and goodness of fit *S* are based on *F*^2^, conventional *R*-factors *R* are based on *F*, with *F* set to zero for negative *F*^2^. The threshold expression of *F*^2^ > σ(*F*^2^) is used only for calculating *R*-factors(gt) *etc*. and is not relevant to the choice of reflections for refinement. *R*-factors based on *F*^2^ are statistically about twice as large as those based on *F*, and *R*- factors based on ALL data will be even larger.

### Fractional atomic coordinates and isotropic or equivalent isotropic displacement parameters (Å^2^)

**Table:**

	*x*	*y*	*z*	*U*_iso_*/*U*_eq_	
Cl1	−0.02192 (8)	0.53784 (7)	0.18886 (5)	0.0355 (2)	
N1	0.3768 (2)	0.1522 (2)	0.49990 (15)	0.0255 (5)	
H1N	0.372 (3)	0.073 (3)	0.4970 (19)	0.026 (7)*	
N2	0.5083 (2)	0.3334 (2)	0.53797 (15)	0.0288 (5)	
N3	0.6177 (2)	0.13385 (19)	0.51466 (14)	0.0252 (5)	
N4	0.1456 (2)	0.1274 (2)	0.53239 (15)	0.0271 (5)	
H4N	0.100 (3)	0.085 (3)	0.490 (2)	0.026 (7)*	
N5	0.1916 (2)	0.1776 (2)	0.68744 (15)	0.0297 (5)	
N6	0.0426 (2)	0.0032 (2)	0.62930 (15)	0.0324 (5)	
C1	0.2469 (3)	0.2170 (2)	0.51066 (17)	0.0236 (5)	
H1A	0.2701	0.2771	0.5635	0.028*	
C2	0.1814 (3)	0.2927 (2)	0.42613 (17)	0.0231 (5)	
C3	0.2361 (3)	0.2959 (2)	0.34541 (18)	0.0281 (6)	
H3A	0.3172	0.2468	0.3403	0.034*	
C4	0.1729 (3)	0.3708 (3)	0.27187 (17)	0.0305 (6)	
H4A	0.2106	0.3727	0.2168	0.037*	
C5	0.0551 (3)	0.4423 (2)	0.27932 (17)	0.0257 (5)	
C6	−0.0013 (3)	0.4405 (2)	0.35850 (18)	0.0274 (5)	
H6A	−0.0824	0.4898	0.3633	0.033*	
C7	0.0623 (3)	0.3653 (2)	0.43127 (17)	0.0263 (5)	
H7A	0.0235	0.3635	0.4860	0.032*	
C8	0.5041 (3)	0.2088 (2)	0.51871 (16)	0.0227 (5)	
C9	0.6373 (3)	0.3841 (3)	0.5586 (2)	0.0346 (6)	
H9A	0.6452	0.4713	0.5741	0.041*	
C10	0.7596 (3)	0.3161 (3)	0.5585 (2)	0.0353 (7)	
H10A	0.8505	0.3537	0.5745	0.042*	
C11	0.7431 (3)	0.1905 (3)	0.53381 (18)	0.0295 (6)	
H11A	0.8254	0.1421	0.5303	0.035*	
C12	0.1273 (3)	0.1023 (3)	0.61980 (17)	0.0266 (5)	
C13	0.1706 (3)	0.1484 (3)	0.77168 (19)	0.0346 (6)	
H13A	0.2127	0.2005	0.8215	0.041*	
C14	0.0906 (3)	0.0461 (3)	0.7899 (2)	0.0406 (7)	
H14A	0.0802	0.0247	0.8507	0.049*	
C15	0.0269 (3)	−0.0232 (3)	0.7149 (2)	0.0406 (7)	
H15A	−0.0309	−0.0929	0.7247	0.049*	

### Atomic displacement parameters (Å^2^)

**Table:**

	*U*^11^	*U*^22^	*U*^33^	*U*^12^	*U*^13^	*U*^23^
Cl1	0.0406 (4)	0.0320 (4)	0.0312 (4)	0.0008 (3)	−0.0014 (3)	0.0066 (3)
N1	0.0227 (11)	0.0213 (11)	0.0316 (12)	−0.0002 (9)	0.0021 (9)	0.0004 (9)
N2	0.0303 (12)	0.0236 (11)	0.0335 (12)	−0.0039 (9)	0.0081 (9)	−0.0030 (9)
N3	0.0245 (11)	0.0236 (11)	0.0263 (11)	−0.0021 (9)	0.0016 (8)	−0.0015 (9)
N4	0.0272 (11)	0.0300 (12)	0.0229 (11)	−0.0074 (9)	0.0016 (9)	−0.0006 (9)
N5	0.0288 (12)	0.0333 (12)	0.0267 (11)	−0.0018 (10)	0.0043 (9)	−0.0002 (9)
N6	0.0310 (12)	0.0394 (13)	0.0256 (11)	−0.0081 (10)	0.0021 (9)	0.0041 (10)
C1	0.0227 (12)	0.0234 (12)	0.0238 (12)	−0.0010 (10)	0.0021 (9)	−0.0017 (10)
C2	0.0237 (12)	0.0221 (12)	0.0235 (12)	−0.0048 (10)	0.0042 (9)	0.0003 (10)
C3	0.0300 (14)	0.0268 (13)	0.0287 (13)	0.0029 (11)	0.0082 (11)	−0.0007 (11)
C4	0.0366 (15)	0.0345 (15)	0.0220 (12)	−0.0018 (12)	0.0098 (11)	−0.0007 (11)
C5	0.0288 (13)	0.0235 (12)	0.0230 (12)	−0.0030 (10)	−0.0001 (10)	−0.0001 (10)
C6	0.0238 (12)	0.0273 (13)	0.0300 (13)	0.0005 (10)	0.0023 (10)	−0.0025 (10)
C7	0.0246 (13)	0.0306 (13)	0.0245 (12)	−0.0010 (11)	0.0065 (10)	−0.0003 (10)
C8	0.0261 (13)	0.0221 (12)	0.0199 (12)	−0.0036 (10)	0.0039 (10)	0.0014 (10)
C9	0.0353 (15)	0.0254 (13)	0.0458 (16)	−0.0095 (12)	0.0147 (13)	−0.0094 (12)
C10	0.0306 (14)	0.0339 (15)	0.0437 (16)	−0.0109 (12)	0.0128 (12)	−0.0118 (13)
C11	0.0267 (13)	0.0306 (14)	0.0316 (14)	−0.0019 (11)	0.0065 (11)	−0.0009 (11)
C12	0.0225 (12)	0.0311 (14)	0.0256 (13)	0.0013 (11)	0.0028 (10)	0.0023 (11)
C13	0.0317 (15)	0.0410 (16)	0.0291 (14)	0.0026 (12)	0.0004 (11)	−0.0044 (12)
C14	0.0368 (16)	0.059 (2)	0.0258 (14)	−0.0061 (15)	0.0058 (12)	0.0057 (14)
C15	0.0346 (16)	0.0541 (19)	0.0330 (15)	−0.0126 (14)	0.0059 (12)	0.0119 (14)

### Geometric parameters (Å, °)

**Table:**

Cl1—C5	1.732 (3)	C3—C4	1.394 (4)
N1—C8	1.345 (3)	C3—H3A	0.9500
N1—C1	1.456 (3)	C4—C5	1.381 (4)
N1—H1N	0.84 (3)	C4—H4A	0.9500
N2—C9	1.334 (3)	C5—C6	1.376 (4)
N2—C8	1.347 (3)	C6—C7	1.388 (4)
N3—C11	1.329 (3)	C6—H6A	0.9500
N3—C8	1.358 (3)	C7—H7A	0.9500
N4—C12	1.362 (3)	C9—C10	1.377 (4)
N4—C1	1.435 (3)	C9—H9A	0.9500
N4—H4N	0.83 (3)	C10—C11	1.378 (4)
N5—C13	1.334 (4)	C10—H10A	0.9500
N5—C12	1.339 (3)	C11—H11A	0.9500
N6—C15	1.331 (4)	C13—C14	1.381 (4)
N6—C12	1.349 (3)	C13—H13A	0.9500
C1—C2	1.523 (3)	C14—C15	1.378 (4)
C1—H1A	1.0000	C14—H14A	0.9500
C2—C3	1.389 (3)	C15—H15A	0.9500
C2—C7	1.390 (3)		
			
C8—N1—C1	122.2 (2)	C5—C6—H6A	120.6
C8—N1—H1N	120 (2)	C7—C6—H6A	120.6
C1—N1—H1N	116 (2)	C6—C7—C2	121.7 (2)
C9—N2—C8	115.6 (2)	C6—C7—H7A	119.2
C11—N3—C8	115.6 (2)	C2—C7—H7A	119.2
C12—N4—C1	123.3 (2)	N1—C8—N2	118.0 (2)
C12—N4—H4N	118 (2)	N1—C8—N3	116.1 (2)
C1—N4—H4N	119 (2)	N2—C8—N3	125.9 (2)
C13—N5—C12	115.7 (2)	N2—C9—C10	123.2 (3)
C15—N6—C12	115.8 (2)	N2—C9—H9A	118.4
N4—C1—N1	110.0 (2)	C10—C9—H9A	118.4
N4—C1—C2	109.5 (2)	C9—C10—C11	116.5 (3)
N1—C1—C2	113.2 (2)	C9—C10—H10A	121.8
N4—C1—H1A	108.0	C11—C10—H10A	121.8
N1—C1—H1A	108.0	N3—C11—C10	123.1 (3)
C2—C1—H1A	108.0	N3—C11—H11A	118.4
C3—C2—C7	118.4 (2)	C10—C11—H11A	118.4
C3—C2—C1	123.7 (2)	N5—C12—N6	126.1 (2)
C7—C2—C1	117.9 (2)	N5—C12—N4	118.2 (2)
C2—C3—C4	120.4 (2)	N6—C12—N4	115.7 (2)
C2—C3—H3A	119.8	N5—C13—C14	123.1 (3)
C4—C3—H3A	119.8	N5—C13—H13A	118.5
C5—C4—C3	119.8 (2)	C14—C13—H13A	118.5
C5—C4—H4A	120.1	C15—C14—C13	116.2 (3)
C3—C4—H4A	120.1	C15—C14—H14A	121.9
C6—C5—C4	120.9 (2)	C13—C14—H14A	121.9
C6—C5—Cl1	119.1 (2)	N6—C15—C14	123.0 (3)
C4—C5—Cl1	120.0 (2)	N6—C15—H15A	118.5
C5—C6—C7	118.8 (2)	C14—C15—H15A	118.5
			
C12—N4—C1—N1	96.1 (3)	C1—N1—C8—N3	173.6 (2)
C12—N4—C1—C2	−139.0 (2)	C9—N2—C8—N1	178.2 (2)
C8—N1—C1—N4	−150.6 (2)	C9—N2—C8—N3	−3.1 (4)
C8—N1—C1—C2	86.6 (3)	C11—N3—C8—N1	−179.7 (2)
N4—C1—C2—C3	−120.7 (3)	C11—N3—C8—N2	1.6 (4)
N1—C1—C2—C3	2.5 (3)	C8—N2—C9—C10	1.5 (4)
N4—C1—C2—C7	61.2 (3)	N2—C9—C10—C11	1.2 (4)
N1—C1—C2—C7	−175.7 (2)	C8—N3—C11—C10	1.5 (4)
C7—C2—C3—C4	0.3 (4)	C9—C10—C11—N3	−2.8 (4)
C1—C2—C3—C4	−177.9 (2)	C13—N5—C12—N6	1.4 (4)
C2—C3—C4—C5	0.0 (4)	C13—N5—C12—N4	−179.7 (2)
C3—C4—C5—C6	−0.2 (4)	C15—N6—C12—N5	−2.5 (4)
C3—C4—C5—Cl1	178.8 (2)	C15—N6—C12—N4	178.6 (3)
C4—C5—C6—C7	0.1 (4)	C1—N4—C12—N5	10.1 (4)
Cl1—C5—C6—C7	−179.0 (2)	C1—N4—C12—N6	−170.9 (2)
C5—C6—C7—C2	0.3 (4)	C12—N5—C13—C14	1.4 (4)
C3—C2—C7—C6	−0.4 (4)	N5—C13—C14—C15	−2.8 (5)
C1—C2—C7—C6	177.8 (2)	C12—N6—C15—C14	0.8 (5)
C1—N1—C8—N2	−7.6 (3)	C13—C14—C15—N6	1.6 (5)

### Hydrogen-bond geometry (Å, °)

**Table:**

*D*—H···*A*	*D*—H	H···*A*	*D*···*A*	*D*—H···*A*
N1—H1N···N3^i^	0.84 (3)	2.20 (3)	3.033 (3)	174 (3)
N4—H4N···N6^ii^	0.83 (3)	2.24 (3)	3.057 (3)	172 (3)

Symmetry codes: (i) −*x*+1, −*y*, −*z*+1; (ii) −*x*, −*y*, −*z*+1.
